# Using Mobile Phones for Adolescent Research in Low and Middle Income Countries: Preliminary Findings From the Birth to Twenty Cohort, South Africa

**DOI:** 10.1016/j.jadohealth.2009.09.008

**Published:** 2010-03

**Authors:** Alastair C. van Heerden, Shane A. Norris, Linda M. Richter

**Affiliations:** a Child Family Youth and Social Development, Human Sciences Research Council, Pietermaritzburg, South Africa; b Birth to Twenty Research Programme, Department of Paediatrics, University of the Witwatersrand Johannesburg, South Africa; c Department of Paediatrics, University of Cambridge, Cambridge, UK

**Keywords:** Mobile phone, Data collection

## Abstract

Mobile phones enable engagement with adolescents through a familiar medium. Survey data are presented from 2,023 South African adolescents who were asked about phone ownership, usage, and their willingness to divulge sensitive information in short message service surveys. Barriers to participation are addressed as are recommendations for follow-up research.

From fixed line telephones as an interview medium in the 1950s to more recent computer-aided surveys, technological innovation brings new opportunities. Mobile phones (MPs) offer potential benefits for research data collection, particularly surveys aimed at adolescents. The International Telecommunications Union reports that < 1 billion new subscriptions were added during 2008, bringing the worldwide total to 4 billion; approximately one-third of these in Brazil, Russia, India, and China [1].

SexINFO [2] was the first mobile service in the United States offering sexual health information directly to adolescents. In response to an opt-in text message, a menu of questions is returned with instructions to text, for example, “D2 if u think ur pregnant.” A similar concept allows youth in South Africa to locate the nearest HIV testing centre by texting “HIV” and their town name to a 5-digit number [3]. A Cape Town-based study piloted the use of MPs as a means for sending daily treatment reminders to tuberculosis patients on a DOTS (directly observed treatment, short-course) program. This decreased regular workload of DOTS employees and gave them sufficient time to focus on noncompliant patients. Of the 300 patients on the program, the World Health Organization reports only five treatment failures and now recommend this model as one of best practice [4]. These examples illustrate the potential of mobile devices. We investigated the viability of delivering self-administered surveys to adolescents on their MPs.

## Methods

Birth to Twenty (BT20) is a birth cohort study based in Soweto-Johannesburg; 3,273 newborns and their mothers were enrolled in 1990 to track health and development over the first 20 years of life [5]. Data are collected every 6 months through face-to-face and computer-assisted self-interviews. Plans are underway to introduce a MP data collection platform. The rationale includes increasing the ecological validity and improving the reliability and accuracy of sensitive self-report data. A telephonic survey to determine acceptability and feasibility of using MPs was conducted with BT20 participants, 2,023 of whom responded (88% of current participants; 52% female; 79% black). Each interview was approximately 5 minutes long, with the major reason for nonparticipation being unavailability at the time of call. The survey was completed by 2,023 participants (88% of current participants, 52% female, 79% black). Despite adolescents were mostly living in households that had electricity (79%), indoor running water (69%), and indoor sanitation (54%), these households were considered poor relative to high-income country households. Questions were designed to assess the current perceptions and attitudes of BT20 youth to MP ownership and use in preparation for research use. The survey, an integral part of BT20, received human subject research approval from the University of the Witwatersrand. Pearson chi-square tests, frequency tables, and other descriptive techniques were used to analyze the data.

## Results

Among respondents, 69% reported currently owning a MP. By contrast, only 43% had access to a landline at home. Approximately 66% of the 31% reported feeling socially isolated as a result of not owning their own MP.

No gender difference was found in how adolescents choose their phone; 60% selected on functionality, followed by 38% who made the decision on the basis of styling. Only 8% chose a phone primarily on the basis of price. There were significant differences (*χ^2^ = *62.26,* df = *3,* p < *.01**) in phone ownership by ethnic group. Significantly, more white adolescents (95%) owned a mobile as compared with black (67%) and colored (63%) adolescents. Most of the participants (86.3%) reported being on a pay-as-you go plan. Of those on pay-as-you go, 62.1% paid for their own airtime and the remaining one-third received airtime from their parents.

Table 1 provides a summary of most valued MP features. There are few clear gender differences; females placed high value on an MP being able to play music. The most used MP features were listening to music, texting, and taking photographs.

When asked which medium they would prefer to answer sensitive questions, 73.1% chose face-to-face interviews over MP surveys. However, Table 2 highlights an interesting pattern between interview preference and knowledge and use of telecommunication technologies. There was a significant likelihood that those who had used Mxit, a popular mobile instant messaging application, or knew or used Skype, a voice-over-IP application, would prefer a MP questionnaire over the conventional face-to-face interview.

## Discussion

Three main concerns emerged regarding using MPs to collect data. These were: (a) not having airtime with which to respond, (b) lacking confidence about understanding the questions being asked, and (c) issues of confidentiality about who might see their answers.

Despite the advantages of speed, event proximity, and logistical ease, it is necessary to address these issues. First, airtime can be considered whenever surveys are administered. Second, questions must be worded clearly with detailed instructions prefacing each section. Finally, a methodology that ensures participant privacy and allows for consent to be obtained using the handset is imperative. Although not relevant to this cohort, the practice of handset sharing could affect negatively on privacy. It is not possible to password protect an SMS. Therefore, if information is sent by the researcher using this protocol it may compromise on the privacy of the participants. This challenge may be largely resolved by mobile survey software that can be password protected and by encrypting data.

Knowing that adolescent's value multimedia content on their MPs raises the possibility of using popular songs, ringtones, and screensaver downloads as an incentive for completing a survey.

Although low literacy rates and language barriers can be a challenge for self-completed questionnaires, providing the survey in colloquial languages is technologically simple on MPs. Each participant could receive the survey in their language of choice.

MPs provide an important platform by which to engage adolescents. The devices have penetrated even the most rural villages, are highly valued by adolescents [6], and offer instantaneous bidirectional communication. How to best scale these projects, manage informed consent, and maintain privacy are all issues which need urgent attention as technological advancements continue to push the bounds of what is currently possible.

## Figures and Tables

**Table 1:** Importance of mobile phone feature by gender

	Males	Females
Making voice calls	284 (64.3%)	280 (60.1%)
Sending SMSs	294 (66.5%)	313 (67.0%)
Sending MMS	228 (51.7%)	252 (54.1%)
Listening to music	299 (67.8%)^a^	350 (74.9%)^b^
Having a camera	274 (62.3%)	303 (65.0%)
Is able to connect to the Internet	224 (50.9%)	239 (51.3%)
Being able to do mobile banking	50 (11.4%)	53 (11.4%)

a Significantly less than expected (*p* < .05).

b Significantly more than expected (*p* < .05).

**Table 2:** Interview preference and technological affinity

	Response	Mobile phone questionnaire	Face-to-face interview
Do you know what Mxit is?	No	43 (13.0%)	119 (13.3%)
	Yes	287 (87.0%)	777 (86.7%)
Have you ever used Mxit?	No	110 (36.9%)^a^	358 (43.9%)^b^
	Yes	188 (63.1%)^b^	457 (56.1%)^a^
Do you know what Skype is?	No	275 (83.8%)^a^	801 (89.9%)^b^
	Yes	53 (16.2%)^b^	90 (10.1%)^a^
Have you ever used Skype?	No	110 (84.0%^a^	300 (91.7%)^b^
	Yes	21 (16.0%)^b^	27 (8.3%)^a^

a Significantly more than expected (*p* < .05).

b Significantly more than expected (*p* < .05).
